# Epstein–Barr Virus Detection in the Central Nervous System of HIV-Infected Patients

**DOI:** 10.3390/pathogens11101080

**Published:** 2022-09-22

**Authors:** Kalo Musukuma-Chifulo, Omar Khalik Siddiqi, Obvious Nchimunya Chilyabanyama, Matthew Bates, Caroline Cleopatra Chisenga, Michelo Simuyandi, Edford Sinkala, Xin Dang, Igor Jerome Koralnik, Roma Chilengi, Sody Munsaka

**Affiliations:** 1 Department of Biomedical Science, School of Health Sciences, University of Zambia, Lusaka P.O. Box 50110, Zambia; 2 Department of Research, Centre for Infectious Disease Research in Zambia, Lusaka P.O. Box 34681, Zambia; 3 Global Neurology Program, Department of Neurology, Beth Israel Deaconess Medical Center, Boston, MA 02215, USA; 4 Department of Internal Medicine, Center for Virology and Vaccines Research, Beth Israel Deaconess Medical Center, Boston, MA 02215, USA; 5 Department of Internal Medicine, School of Medicine, University of Zambia, Lusaka P.O. Box 50110, Zambia; 6 School of Life & Environmental Sciences, University of Lincoln, Lincoln LN6 7TS, UK; 7 HerpeZ Infection Research and Training, University Teaching Hospital, Lusaka Private Bag RW1X Ridgeway, Lusaka P.O. Box 10101, Zambia; 8 Department of Neurology, Northwestern University Feinberg School of Medicine, Chicago, IL 60611, USA

**Keywords:** Epstein–Barr virus (EBV), central nervous system (CNS), human immunodeficiency virus (HIV), polymerase chain reaction (PCR), cerebrospinal fluid (CSF)

## Abstract

Simply detecting Epstein–Barr virus deoxyribonucleic acid (EBV-DNA) is insufficient to diagnose EBV-associated diseases. The current literature around EBV-DNA detection from cerebrospinal fluid (CSF) in human immunodeficiency virus (HIV)-positive non-lymphoma patients was systematically reviewed and a meta-analysis reporting the estimated pooled prevalence in this population when PCR methods are employed, targeting different sequence segments within the EBV genome, was conducted. Using a combination of three key concepts—Epstein–Barr virus detection, central nervous system disease, and human cerebrospinal fluid—and their MeSH terms, the PubMed database was searched. A total of 273 papers reporting the detection of EBV in CNS were screened, of which 13 met the inclusion criteria. The meta-analysis revealed a pooled prevalence of EBV-DNA in CSF of 20% (CI: 12–31%). The highest pooled prevalence was from studies conducted on the African population at 39% (CI: 27–51%). The investigation of the presence of EBV-DNA in the CSF was also very varied, with several gene targets used. While most patients from the articles included in this review and meta-analysis were symptomatic of CNS disorders, the pathogenicity of EBV in non-lymphoma HIV patients when detected in CSF has still not been determined. The presence of EBV-DNA in the CNS remains a concern, and further research is warranted to understand its significance in causing CNS disorders.

## 1. Introduction

Epstein–Barr Virus (EBV) is a herpes virus and has infected over 90% of the population of the world [1,2,3,4,5,6,7]. With primary infection occurring in the tonsillar tissues [8,9], EBV infection is persistent and life-long as the virus goes into latency in B-cells [10,11,12,13,14]. It is controlled by EBV-specific cytotoxic CD8^+^ T-lymphocytes [1,2,13,15,16,17]. There is a balance between the cells latently infected with the EBV genome and those undergoing lytic infection [8]. However, when the immune system is compromised, as may occur in the presence of HIV, this balance is lost, and EBV may enter a more actively replicating state [11]. Uncontrolled EBV replication may lead to central nervous system (CNS) infection.

Data have shown an impairment to CD8+ T-cell surveillance in HIV-infected patients, which renders EBV-specific immune control less efficient in addition to the reduction in their count [11,18]. This impaired immunity predisposes individuals to developing other pathogens that may persist [5,18,19]. The introduction of HAART after 1995 [19] tremendously reduced the risk of HIV-infected patients developing EBV-related malignancies due to improved immune surveillance and T-cell immunity [20,21,22]. While this is true, frequent activation of the EBV is still a common phenomenon [20], and infection of the CNS with EBV in HIV-positive patients is associated with reactivated EBV [23]. It is thought that under these circumstances, EBV occurs in the CNS as an opportunistic infection [18,21]. EBV-DNA in the CNS is often detected together with other pathogens but more frequently than others, especially in patients with suspected meningitis and with HIV [24,25,26]. Currently, limited data have been shown on the presence of EBV-DNA in meningitis and indeed in the absence of lymphoma.

To date, there is no approved treatment for EBV [27]. Acyclovir (ACV) is one of the drugs that exerts antiviral activity against EBV by blocking viral replication. It reduces shedding in the oropharynx but has no clinical benefit. Ganciclovir also has antiviral properties and is more effective than ACV but more toxic. It should be noted that these drugs only affect the virus as it replicates; hence, they cannot affect EBV in latency as the virus would have formed an episome [28]. Apart from antiviral drugs, immunogens are sometimes used in the treatment of EBV. These are used to induce T-cells responses, and suggestions have been made for their use as prophylactic EBV vaccines [29]. Immunomodulatory agents such as interferon alpha (IFN-a) and interleukin 2 (IL-2) have been used to treat chronic active EBV with limited success and to treat EBV-associated malignancies. The goal is to enhance T-cell-mediated immunity to EBV proteins expressed in the tumour cells, especially EBV EBNAs and LMPs [28]. 

The current laboratory diagnostic test of CNS EBV disease is DNA PCR testing on cerebrospinal fluid (CSF) [24,30,31]. Given its increased sensitivity compared to the traditional gold standard of culture, this method is widely adopted in this field as it also offers more reliable results [32,33]. Nonetheless, because EBV results in the lifelong infection of B cells, the detection of EBV-DNA does not necessarily indicate the presence of active disease [15,34]. 

We systematically reviewed the current literature around EBV-DNA detection from the CSF of HIV patients with non-lymphoma clinical disease. 

## 2. Results

### 2.1. Selection of Articles

A total of 273 articles were returned from the PubMed search, and ten additional articles were identified through Google search and reference lists from the identified articles. A total of 13 articles were finally included in this review. We followed the PRISMA 2009 guidance for systematic reviews for the screening, eligibility, and selection of articles. Two hundred and fifty nine articles were initially excluded as follows: review articles (*n* = 25), case reports (*n* = 99), organ recipients (*n* = 16), EBV studied in non-humans (*n* = 6), articles not in English (*n* = 15), assay development (*n* = 26), studies not focused on EBV (*n* = 35), recommendations on EBV management (*n* = 4), EBV detection not from CSF (*n* = 9), HIV status not clearly stated (*n* = 16), patients diagnosed with lymphoma (*n* = 10), EBV detected at autopsy (*n* = 3), and sub-analysis from an already included study (*n* = 1). 

**Figure 1:**
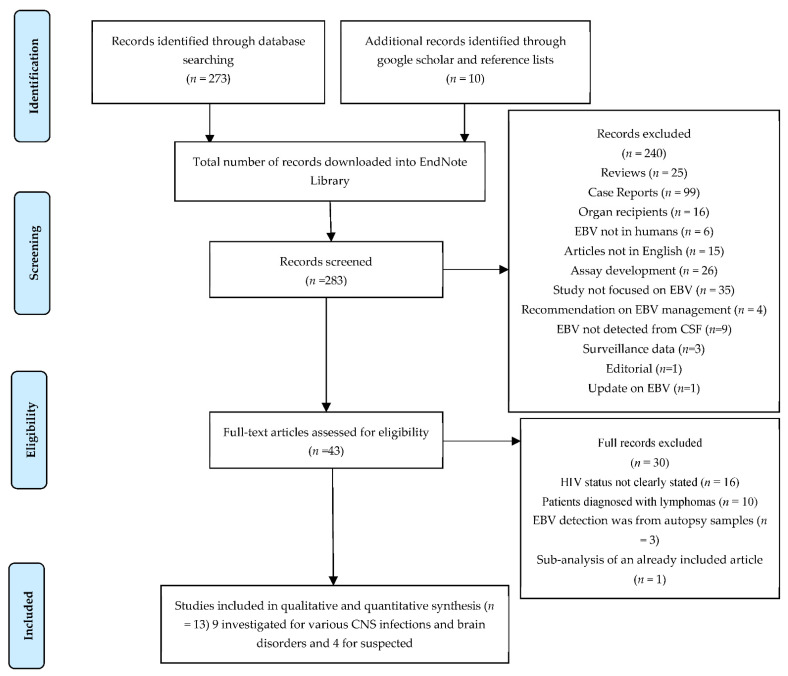
PRISMA 2009 flow diagram of article identification, screening, eligibility, and inclusion.

Figure 1 below was generated to summarise the process.

### 2.2. Quality Assessment 

Only one of the 13 articles included in this review was ranked as moderate quality [35], with all the other studies ranked as high quality. The average percentage score of all the studies was 84%. One notable poorly scored question on the checklist was that of clearly describing the inclusion and exclusion criteria. Only three studies satisfied this criterion, with nine studies only extensively describing the inclusion criteria [36,37,38]. All the other scores are summarised in Table 1 below.

### 2.3. Characteristics of Studies Included in the Review

This review included 13 studies conducted between 1998 and 2020. Most studies were from Africa (*n* = 5) and Europe (*n* = 5), and others were from Asia (*n* = 2) and South America (*n* = 1). Five of the thirteen (38%) studies were retrospective, and another three (23%) were prospective. Two of the studies (15%) had a cross-sectional design, while only one was a hospital-based study (8%) (Table 1). Only two (15%) of the included studies focused on EBV alone, while nine (69%) focused on the different human herpes viruses, including EBV. Three of the thirteen studies (23%) focused on any eatiologies of CNS diseases.

The characteristics of the articles included in this systematic review and meta-analysis are summarised below (Table 2).

### 2.4. Participant Characteristics

In seven (54%) of the articles, the patients presented with signs indicative of meningitis (headache, stiff neck, confusion altered mental status, vomiting, and photophobia). Five (38%) studies reported that patients showed signs of infection such as fever, while others reported opportunistic infections. Four (31%) of the studies included patients with signs of meningoencephalitis. Five (38%) studies reported on patients showing signs of peripheral nerve defects, new and persistent neurological symptoms, worsening cognitive impairment, generalised or partial seizures, and the need for lumbar puncture (LP) on the assumption of syphilis or white matter hyperintensity on an MRI. 

### 2.5. Laboratory Diagnosis of EBV

All the studies included in this review screened for EBV in CSF using PCR. Different laboratories used various DNA extraction methods, mainly in-house, on a wide range of CSF volumes. A few commercial extraction methods such as QIAGEN and EasyMag were used. The DNA amplification methods were predominantly in-house-developed procedures. The detection methods also varied and included gel electrophoresis, Southern blotting, and target-specific probes. 

The different EBV target regions used to detect EBV-DNA included EBNA-1, BamH1-W, BNRF 1 gene, BNFR1 p143 gene, EBV IR, LMP 2 gene, the Sma1 and BamH1 regions, and BALF5. Five of the studies did not clearly state the EBV target region.

### 2.6. Empirical Treatments 

In two studies, some patients were treated empirically with anti-toxoplasma and, in others, anti-tuberculosis. The drugs included in these empirical treatments were pyrimethamine, sulphadiazine, fluconazole, and ceftriaxone, principally to cover other CNS infections [36,40]. 

### 2.7. Prevalence 

The prevalence of EBV-DNA in the CSF of 1707 samples ranged from 2% to 51%. The highest EBV-DNA positivity was observed in studies conducted from Africa [15,38,39,47]. The lowest positivity was reported in a study from Asia, followed by South America, and finally, Europe.

Generally, all studies from Africa reported higher EBV prevalence. The EBV-DNA loads were reported in only two studies and were reported as normal or abnormal CSF by Kelly et al. (2011), and as final diagnosis by Corcoran et al. (2008) [15,45]. Kelly et al. (2011), reported a higher EBV-DNA load (6849 cp/mL) in patients with abnormal CSF (described as ≥5 white blood cells per mm^3^ in the CSF) compared to the 1202 cp/mL in those with normal CSF (described as <5 white blood cells per mm^3^ in the CSF). In the study by Corcoran et al., where individual-level data were reported, a median EBV-DNA load of 4924 cp/mL in the sixteen non-lymphoma patients was reported.

### 2.8. Meta-Analysis

**Figure 2:**
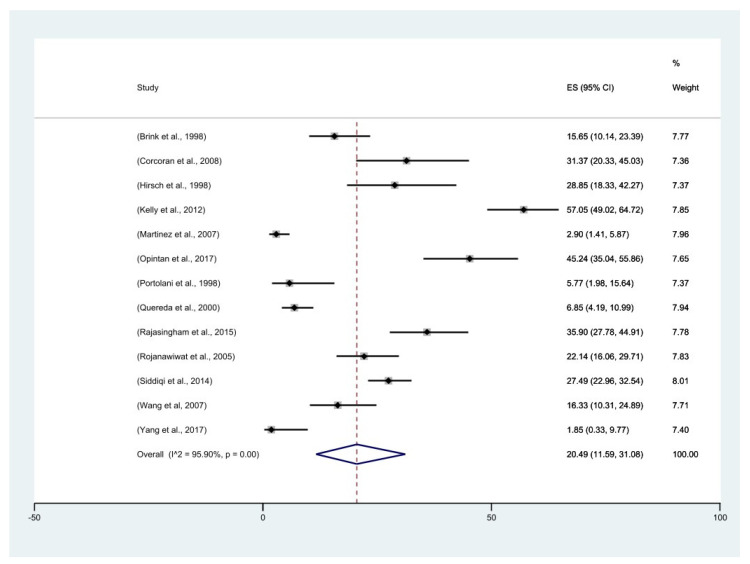
Forest plots of prevalence (%) of EBV−DNA in CSF (*n* = 13 studies) using the random−effects model. At 95% CI, the dashed line represents the overall mean prevalence, the overall pooled estimate is represented by the blue diamond and the point estimates of each study weighted for population size are represented by the black diamonds [15,35,36,37,38,39,40,41,42,43,44,45,46].

**Figure 3:**
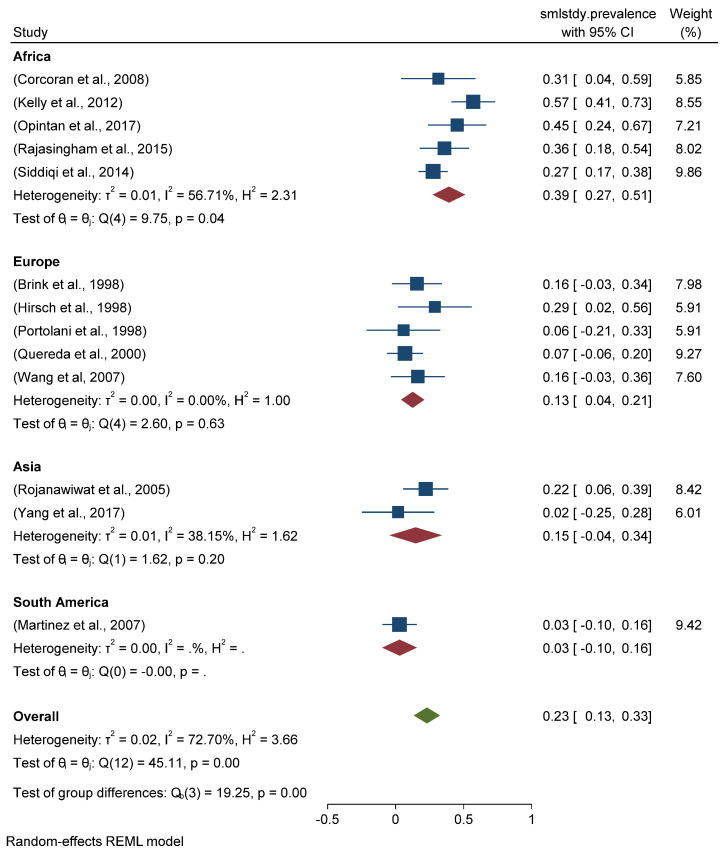
Subgroup analysis−pooled prevalence of EBV−DNA in CSF by region of study. At 95% CI, the overall pooled estimate is represented by the green diamond, the pooled prevalence for each region is represented by the maroon diamonds, and the point estimates of each study weighted for population size are represented by the blue squares [15,35,36,37,38,39,40,41,42,43,44,45,46].

The overall random-effects pooled prevalence for all studies was 20% (CI: 12–31%) with high heterogeneity (I^2^ = 96%, *p* = 0.00) (Figure 2). We explored the heterogeneity between studies using subgroup analysis and meta-regression. One striking result in a subgroup analysis by region showed that the highest pooled prevalence of EBV-DNA in CSF using PCR was from studies conducted in Africa (39%, CI: 27–51%), with the lowest prevalence in studies from South America (3% CI: 0–16%) (Figure 3). Generally, the reported prevalences from African studies were higher (Figure 3). Compared to the overall pooled prevalence regardless of the region, which was 20% (CI: 12–31%), the pooled prevalence when analysed by region was higher (23% (95% CI: 13–33%)). 

**Figure 4:**
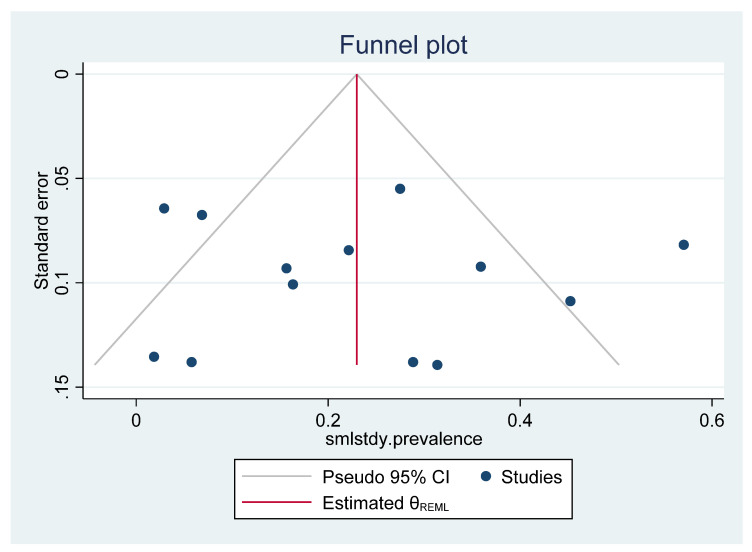
Funnel plot of standard error by sample size and prevalence. Individual studies are represented by small circles (Egger’s test: *p* = 0.9954).

Using the Egger-test, the small-study effect was investigated (Egger-test, *p* = 0.9954). A trim-and-fill analysis using the random-effects model was also performed. The estimates (prevalence) and the errors were scattered in a non-systematic way; therefore, there was no evidence of a small-study effect (Figure 4).

## 3. Discussion

Several reviews have been conducted on the presence of EBV-DNA in the CSF of HIV patients with lymphoma, with various conclusions drawn on its use, especially in PCNSL [25]. However, very few studies have described the presence of EBV-DNA in non-lymphoma HIV patients. 

This review reports an overall pooled prevalence of 20% (CI: 12–31%, overall I^2^: 96%, *p* = 0.00) in HIV-positive non-lymphoma patients. When a sub-analysis of the reported prevalence was conducted by region, Africa had the highest pooled prevalence (39%; 95% CI: 27–51%) compared to Europe (13%; 95% CI: 4–21%), and Asia (15%; 95% CI: 0–34%). This finding points to the likely high burden of EBV-DNA in the CSF of HIV-positive patients in Africa and prompts a call for further investigations. There was also reduced heterogeneity among studies when they were analysed by region. The differences in the reported pooled prevalence by region could be due to previous reports on the distribution of EBV subtypes by region. The most widely spread subtype is A [47,48], with Sub-Saharan Africa; having an equal distribution of both subtypes A and B could be one reason the prevalence is higher in Africa [49,50,51,52,53]. Moreso, EBV subtype 2 is implicated in reports of it being more virulent [54]. A study by Petrara et al. reported a higher viral load from individuals infected with both subtypes, which might be the case for African studies [55]. While we did not have a study in this review that had reported on the EBV variants in their population, we can speculate that infection with both subtypes renders infection to the CNS more probable and common in this population. Two of the five African studies included in this review reported that only 36% and 30% of the patients were on combination antiretroviral therapy (cART) [36,45]. Rajasingham et al., in their study, included patients not yet initiated on cART [37], while Corcoran et al., and Kelly et al., did not state the cART status of the patients included in their studies [15,44]. Though this pattern does not differ from studies conducted elsewhere in this review, we think the increased pooled prevalence in the African population could be due to several factors, including delayed cART initiation and increased exposure to other pathogens. With HIV as a secondary immunodeficiency, it promotes or facilitates the reactivation of the EBV virus [27,56]

Co-infections in EBV seem to play an important role in the success of its reactivation. It seems quite apparent that EBV may take advantage of the replicative machinery available for the other pathogens and can reactivate. There is still no WHO/FDA-approved treatment for EBV [27,57]. With several drug candidates with activity against EBV, there still is no perfect candidate for EBV treatment. While it is essential to control the EBV virus in patients at high risk of the virus reactivating, initiating cART early to help restore the impaired immune surveillance and T-cells is still an important factor in its control. 

We did not analyse the use of EBV-DNA load in non-lymphoma, as only two studies reported the median loads in their sample populations. This shows the few studies that have tried to understand the use of EBV-DNA load in this population compared to its use in PCNSL. The reported medians from the two studies were much lower than what has been reported in lymphoma patients [58]. Given that some high EBV-DNA loads were observed in some patients, Kelly et al. (2011) speculate that EBV can also arise from within the CNS [15]. Studies examining EBV-DNA loads outside of the CNS in individuals with no known EBV-related disease have reported higher EBV-DNA load in HIV-infected patients compared to their non-HIV-infected counterparts. This finding is factual even in individuals initiated on cART. 

As previously reported, EBV infection in the CNS can be one of the opportunistic infections in this population [2]. Most of the studies included in this review were not solely focused on EBV. It was mainly investigated after other clinical investigations had been conducted in those patients, which is reflective of the retrospective nature of most studies *n* = 5 (38%). The prevalence of EBV-DNA in CSF ranged between 2% and 51%. These results provide more evidence on the importance of extensive screening of the CSF for different aetiologies of CNS disease, including EBV, to improve patients’ diagnoses, targeted treatments, and outcomes, and reduce unnecessary empirical treatments. Different studies used different laboratory techniques, from sample collection and processing to the reporting of results. The target region on the EBV genome also varied, with seven different EBV targets used from the 13 articles reviewed. One article used two different genome targets and compared the frequency of detecting the EBV-DNA from the same samples [59]. As shown by Ryan et al. (2004), the sensitivity of EBV detection can be affected by the target region [60]. More so, the comparability of the quantified EBV-DNA using different target regions can become complicated, especially when target regions that repeatedly appear on the EBV genome (Bam H1 and internal repeats) are compared with those that appear only once. However, the repeated regions offer increased sensitivity when the EBV-DNA is in low quantities as the chances of detecting the virus are increased. At the same time, it has been shown that the assay’s sensitivity can be affected by the target region. Thus, an important limitation of this review is that we cannot be sure that the reported prevalence in these studies equally measured the presence of EBV, as we are unclear on how conserved these target regions are. The assay platforms also varied considerably, with some studies using in-house assays whilst others used already-established and more automated platforms for nucleic acid extraction and detection. 

One strength of this review was the inclusion of studies conducted in routine settings that strengthen our review as they represent a more realistic view of how the public health burden affects routine clinical practice. Several inconsistencies were noted in how the prevalence of EBV is reported. This inconsistency resulted in another limitation for this review in that it was not possible to pool the EBV-DNA prevalence in the CSF of different diagnostic categories of CNS diseases; a meta-analysis would yield high CIs. Some studies also indicated that some patients had received prophylaxis treatment, which was mostly anti-toxoplasma and tuberculosis. A detailed look into this showed that empirical treatments could have contributed to the delay in EBV-targeted testing and naturally influenced the reported prevalence or reported viral loads. It was also impossible to ascertain whether the clinical deterioration of patients reported in some studies could be attributed to the infection itself or to the empirical treatments they were placed on. It does, however, seem essential to initiate targeted EBV treatment early. Furthermore, although it is known that individuals with meningitis tend to have a much lower DNA load compared to those without meningeal inflammation [15], there is insufficient evidence on the fundamental interactions for this. 

## 4. Materials and Methods

### 4.1. Literature Search 

Only studies investigating EBV-DNA in the CNS and reported from CSF samples were included. The Boolean search term in the PubMed database was: (Epstein–Barr virus OR EBV) AND (Central Nervous System OR CNS) AND (CerebrospinalFluid OR CSF).

### 4.2. Data Management 

The PubMed database was systematically searched between 25 March 2020 and 30 December 2020, with no restriction on the date of publication to identify articles that reported the prevalence of EBV-DNA on CSF samples. The identified articles were then downloaded and imported into EndNote X7 software (Thomason Reuters, Toronto, ON, Canada), and all duplicates were removed from the library. Rayyan Software (Rayyan Systems, Inc., Cambridge, MA, USA) [61] was used to allocate articles according to the various groups established (included, maybe, or excluded), with all excluded articles labelled with a reason for exclusion. Articles were excluded if: they were case reports, the HIV status of patients was not clearly stated, they were not focused on EBV, they were review articles, they included patients who were organ recipients, the detection of EBV was not from CSF and by PCR, patients included only those with lymphoma, the samples used were from autopsy samples, or they were editorials, updates, recommendations, assay validation methods, and/or not in English. 

### 4.3. Eligibility Criteria

Included in this review and meta-analysis were articles: whose full text was available in English, from a cohort, and that were cross-sectional or retrospective studies. Studies in languages other than English, case reports including patients after organ transplants, laboratory assay validation studies, and studies not conducted on humans were excluded. All these studies detected EBV in CSF using the PCR method. 

### 4.4. Selection Process 

The returned articles were first screened based on their title and abstract, and the decision as to whether they should be included in the review or not was undertaken by two independent reviewers (K.M.-C. and O.K.S.) using the set inclusion criteria as stated above. A review of the full articles followed this, and any differences in opinion were discussed between the two reviewers, with a third reviewer available (S.M), until the reviewers were in concord.

### 4.5. Data Collection 

The reviewers extracted the data into a form created explicitly for this review with the following domains: duration of the study in months, the country where the study was conducted, study design, sample size, study population, method of laboratory detection, study aim/objective, number of EBV-positive samples, EBV-DNA load, age, CD4, EBV target region, year of publication, and author. 

### 4.6. Quality Assessment

An assessment tool adapted from the Newcastle–Ottawa assessment scale (NOS) and other adapted assessment checklists adapted from the NOS was used to assess each included article in this review and meta-analysis. The adapted assessment tool contains 17 questions, with each question assigned a maximum score of one, leading to a maximum score of 17 for each article. The risk of study bias was ranked as high- (75–100%), moderate- (50–74%), or low-quality (<50%). Studies included in this review and meta-analysis fall either in the high- or moderate-quality ranks.

### 4.7. Systematic Review and Meta-Analysis 

Data synthesis comprised of (i) the characteristics of the studies included in the review, (ii) participant characteristics, (iii) the laboratory diagnosis of EBV, and (iv) empirical treatment was conducted. Where possible, descriptive statistics were used. A meta-analysis on EBV-DNA in CSF patients with pooled estimates and reported 95% confidence intervals was performed. 

A sensitivity analysis of the effect of small studies, the region in which the study was conducted, and the population from which the participants were drawn (with HIV, without HIV, with HIV and CNS, and without HIV and CNS) on the pooled prevalence estimates. Using Higgins I2 values, we assessed heterogeneity among the studies. I-squared values of 25%, 50%, and 75% were classified as low, moderate, and high heterogeneity [62].

Statistical analysis was performed using Stata 16 (StataCorp. 2017 Stata Statistical Software: Release 14. College Station, TX, USA). 

## 5. Conclusions

Outside of detection in brain tissue or meninges, it is unclear whether EBV-DNA detection in patients’ CSF, particularly those with neurological symptoms, accurately indicates EBV-associated disease. While its detection using PCR in the CSF from this population is high at 20% (CI: 12–31%), this could just be the amplification of a quiescent virus in most individuals.

## Figures and Tables

**Table 1:** Quality Assessment Score of Articles.

Author	Was the Study on the Detection of Epstein–Barr Virus DNA in the CSF?	Was the Research Problem Clearly Described?	Was the Hypothesis/Aim/Objective of the Study Clearly Described?	Was the Study Design Clearly Reported?	Was the Study Population Clearly Described?	Were the Study Population Inclusion and Exclusion Criteria Clearly Stated?	Was the EBV Screening Procedure Well Described?	Was the EBV Target Gene Clearly Stated?	Was the Sample Size Cleared Stated?	Were the Characteristics of the Patients Included in the Study Clearly Described?	Were The Statistical Tests Used To Assess The Primary Outcomes Well Described?	Were the Main Outcomes to be Measured Clearly Described in the Methods Section?	Were the Main Findings of the Study Clearly Described?	Were the Main Findings Linked to the Study Aims or Objectives?	Were the Study Results Appropriately Interpreted, e.g., in Terms of the Strength of the Evidence, Its Application/Implications, and Causality?	Was a Summary of the Key Outcomes Clearly Stated?	Were the Findings Compared to Other Relevant Studies?	Total	% Score
Brink et al., 1998	Yes	Yes	Yes	Yes	Yes	P *	Yes	Yes	Yes	P *	P *	Yes	Yes	Yes	Yes	Yes	Yes	14	82
Corcoran et al., 2008	Yes	Yes	Yes	Yes	Yes	No	No	Yes	Yes	Yes	Yes	No	Yes	Yes	Yes	Yes	Yes	14	82
Hirsh et al., 1998	Yes	Yes	Yes	Yes	Yes	P *	Yes	Yes	Yes	Yes	No	Yes	Yes	Yes	Yes	Yes	Yes	15	88
Kelly et al., 2012	Yes	Yes	Yes	Yes	Yes	P *	Yes	Yes	Yes	Yes	Yes	Yes	Yes	Yes	Yes	Yes	Yes	16	94
Martinez et al., 2007	Yes	Yes	Yes	Yes	Yes	P *	Yes	!	Yes	Yes	No	Yes	Yes	Yes	Yes	Yes	Yes	14	82
Opintan et al., 2017	Yes	Yes	Yes	Yes	Yes	Yes	P *	!	Yes	Yes	Yes	Yes	Yes	Yes	Yes	Yes	Yes	15	88
Portolani et al., 1998	Yes	P *	Yes	No	No	No	Yes	Yes	Yes	P *	No	Yes	Yes	Yes	Yes	Yes	Yes	11	65
Quereda et al., 2000	Yes	Yes	Yes	Yes	Yes	P *	Yes	!	Yes	Yes	P *	Yes	Yes	Yes	Yes	Yes	Yes	14	82
Rajasingham et al., 2015	Yes	Yes	Yes	Yes	Yes	Yes	No	No	Yes	Yes	Yes	Yes	Yes	Yes	Yes	Yes	Yes	15	88
Rojanawiwat et al., 2005	Yes	Yes	Yes	P*	Yes	P *	Yes	Yes	Yes	Yes	No	Yes	Yes	Yes	Yes	No	Yes	13	76
Siddiqi et al., 2014	Yes	Yes	Yes	Yes	Yes	P *	Yes	Yes	Yes	Yes	Yes	Yes	Yes	Yes	Yes	Yes	Yes	16	94
Wang et al., 2007	Yes	Yes	Yes	Yes	Yes	P *	P *	Yes	Yes	Yes	P *	Yes	Yes	Yes	Yes	Yes	Yes	14	82
Yang et al., 2017	Yes	Yes	P *	Yes	Yes	Yes	Yes	Yes	Yes	Yes	Yes	Yes	Yes	Yes	Yes	Yes	Yes	16	94

Checklist scoring: Yes = 1; no = 0; P * (partially described) = 0; ! (not sure) = 0. % Score: 75%–100% = high quality; 50% to 75% = moderate quality; <50% = low quality.

**Table 2:** Characteristics of studies included in this review reporting the detection of EBV-DNA from CSF.

	Author, Year [Ref]	Country	Study Design	Sample Size	EBV-Positive via Qualitative PCR % (n/n)	EBV-Positive via Quantitative PCR % (n/n)	Conclusions Drawn from Study
	Europe						
1	Hirsh H et al. (1998) [39]	Switzerland	Prospective study and retrospective study	53	29 (15/52)	N/A	In individuals with HIV-1, EBV PCR was not a specific marker for PCL. CSF screening should be performed before any antiprotozoal therapy is given.
2	Brink et al. (1998) [40]	United Kingdom	Prospective study	115	N/A	16 (18/115)	In HIV, the detection of EBV in CSF is strongly associated with primary CNS lymphoma, while its detection in non-lymphoma patients may predict for subsequent tumour development.
3	Wang et al. (2007) [41]	United Kingdom	Retrospective analysis study	98	16 (16/98)	N/A	In non-lymphoma patients, the significance of EBV detected is unclear and the study suggests that it does not invariably lead to PCL.
4	Portolani et al. (1998) [35]	Italy	Retrospective analysis study	52	6 (3/52)	N/A	Three EBV-positive patients were identified—one from a meningitis patient and two from encephalitis patients.
5	Quereda et al. (2000) [42]	Spain	Retrospective analysis study	219	7 (15/219)	N/A	In the evaluation of herpesviruses-related neurological diseases in HIV-infected patients, using multiplex herpes virus-PCR performed on CSF is a proper diagnostic technique.
	**Asia**						
6	Rojanawiwat et al. (2005) [43]	Thailand	Prospective study	140	22 (31/140)	N/A	The detection of EBV in the CNS in advanced HIV-infected patients in northern Thailand is common.
7	Yang et al. (2017) [38]	China	Hospital-based	54	2 (1/54)	N/A	In meningitis patients, the most common pathogen causing meningitis was *Cryptococcus neoformans*, and the most common viral pathogen causing meningitis was CMV while TB was the most common bacteria.
	**South America**						
8	Martinez et al. (2007) [44]	Cuba	Cross-sectional	241	3 (7/241)	N/A	The use of HAART did not exclude herpesviruses as pathogens involved in the CNS disease.
	**Africa**						
9	(Corcoran et al. (2008) [45]	South Africa	Retrospective study	55	N/A	36 (20/55)	Detection of EBV in CSF of HIV patients with various neurological diseases was common and was poorly predictive of PCNSL, while the absence of EBV-DNA in CSF could reliably be used to exclude PCSNL. The use of a quantitative assay improved the diagnostic specificity.
10	Opintan et al. (2017) [36]	Ghana	Prospective cohort study	84	45 (38/84)	N/A	The use of PCR for the detection of co-infections in CSF revealed what would otherwise remain undetected by routine diagnostics. The high prevalence of CSF co-infection also suggests severe immune suppression.
11	Rajasingham et al. (2015) [37]	Uganda	Prospective cohort study	314	36 (42/117)	N/A	To distinguish between CNS lymphoma and non-pathogenic presence of EBV, quantifying EBV-DNA in CSF and serum may be helpful.
12	Kelly et al. (2011) [15]	Malawi	Retrospective analysis study	188	N/A	45 (85/188)	The study hypothesised that EBV can arise from within the CNS, indicated by the high EBV-DNA loads observed in some patients. The DNA load of EBV can be associated with outcome and its presence in CSF can play a causative role.
13	Siddiqi et al. (2014) [46]	Zambia	Cross-sectional	331	27 (91/331)	N/A	There was a high prevalence of EBV-DNA. EBV-DNA was mostly present together with other pathogens.

Abbreviations: PCL—primary cerebral lymphoma; HIV-1—Human Immunodeficiency Virus-1; PCR—polymerase chain reaction; CSF—cerebrospinal fluid; CNS—central nervous system; CMV—cytomegalovirus; TB—tuberculosis; HAART—highly active antiretroviral therapy; PCNSL—primary central nervous system lymphoma; DNA—deoxyribonucleic acid; EBV-DNA—Epstein–Barr virus deoxyribonucleic acid.

## Data Availability

The data are contained within the article.
